# Operation for Hernia

**Published:** 1861-09

**Authors:** 


II. *Operation for Hernia.*—W. G., a native of France, æt 30, of temperate habits, was admitted into the Marine Hospital, June 3d. The patient has been subject to periosteal pains which yielded to the use of iodide of potassium, and was also afflicted with hernia, an oblique inguinal, for which he sought relief. The tumor instantly appeared upon the patient's assuming the erect position, and readily disappeared in the recumbent. The size of the tumor prevented it being easily and perfectly trussed, and the patient requested that something be offered for the complete cure.

Operation, July 20. The patient was placed under the influence of chloroform, and the surgeon, Dr. Isham, with the left index finger, invaginated the scrotum through the inguinal canal, pushing it quite through the internal abdominal ring, secured the invaginated portion of the scrotum in its new position, by passing along the radial and inner borders of the finger, two long needles armed with a bundle of thread sufficiently large to fill up the punctures made, and threaded upon which, like a bead, was a rounded piece of cork of a proper size to fit into the ring lined by the scrotum. This plug was drawn up and secured, the end of the suture being brought out upon the surface bordering the inner and outer borders of the ring, and tied over a compress. This was suffered to remain for forty hours, with water dressings, when it was removed, and the adhesions were perfect. The scrotum was sustained in a suspensory bandage, and the 22d the patient pronounced cured.

